# Supplementary material for: Predation and fragmentation portrayed in the statistical structure of prey time series
Source: BMC Ecol. 2009 May 6;9:10. doi: 10.1186/1472-6785-9-10 (PMC2689204; doi:10.1186/1472-6785-9-10)
Supplement: Additional file 2 — Voles and related classes ODDox Documentation. ODDox documentation of the agent-based model (ALMaSS) applied by Hendrichsen et al. The documentation is started by activating main.html. [file 1472-6785-9-10-S2.zip › Vole_ODDox/setaside_8h.html]

ALMaSS ODDox: setaside.h File Reference

- Main Page
- Related Pages
- Classes
- Files

# setaside.h File Reference

---

## Detailed Description

**Setaside.h This file contains the headers for the setaside class**   

by Chris J. Topping   
Version of June 2003   
All rights reserved.   
  
With additions as noted in:   
Doxygen formatted comments in July 2008   

|  |  |
| --- | --- |
|  |  |
| Classes | |
| class | SetAside |
|  | Rotational set-aside management class   . More... |
| Defines | |
| #define | SETASIDE\_BASE   600 |
| Enumerations | |
| enum | SetAsideToDo {     sa\_start = 1, sa\_cut\_to\_hay = SETASIDE\_BASE, sa\_cattle\_out, sa\_cattle\_is\_out,     sa\_cut\_to\_silage, sa\_glyphosate, sa\_wait   } |

---

## Define Documentation

#define SETASIDE\_BASE   600

---

## Enumeration Type Documentation

enum SetAsideToDo

Below is the list of things that a farmer can do if he is has rotational setaside. For setaside this list is pretty short, because he can't do much with standard setaside (rules as of DK, 2000).

**Enumerator:**
:   |  |  |
    | --- | --- |
    | *sa\_start* |  |
    | *sa\_cut\_to\_hay* |  |
    | *sa\_cattle\_out* |  |
    | *sa\_cattle\_is\_out* |  |
    | *sa\_cut\_to\_silage* |  |
    | *sa\_glyphosate* |  |
    | *sa\_wait* |  |

```
00058              {
00059   sa_start = 1, // Compulsory, start event must always be 1 (one).
00060   sa_cut_to_hay = SETASIDE_BASE,
00061   sa_cattle_out,
00062   sa_cattle_is_out,
00063   sa_cut_to_silage,
00064   sa_glyphosate,
00065   sa_wait
00066 } SetAsideToDo;
```

---

Generated on Thu Jan 22 14:13:45 2009 for ALMaSS ODDox by 
 1.5.6
